# Cholestéatome de l'oreille moyenne - étude rétrospective à propos de 145 cas

**DOI:** 10.11604/pamj.2014.17.163.3670

**Published:** 2014-03-06

**Authors:** Brahim Bouaity, Mehdi Chihani, Karim Nadour, Mountassir Moujahid, Mliha Touati, Youssef Darouassi, Haddou Ammar

**Affiliations:** 1 Service d'Oto-rhino-laryngologie et Chirurgie Cervico-faciale, Hôpital Militaire Avicenne, Marrakech, Maroc; 2 Service de Chirurgie Générale, Hôpital Militaire Avicenne, Marrakech, Maroc

**Keywords:** Cholestéatome, oreille moyenne, otite cholestéatomateuse, tympanoplastie, cholesteatoma, middle ear, cholesteatoma otitis, tympanoplasty

## Abstract

L'otite moyenne chronique cholestéatomateuse représente une entité fréquente et dangereuse en chirurgie otologique, mettant en jeu le pronostic fonctionnel auditif et exposant à des complications redoutables justifiant pleinement le recours exclusif à un traitement chirurgical et à un suivi post-opératoire rigoureux. Dans le but de mettre le point sur les caractéristiques épidémiologiques, cliniques, thérapeutiques et évolutives de cette pathologie, nous avons mené une étude rétrospective, étalée sur 11 ans et portant sur 145 cas de cholestéatome de l'oreille moyenne opérés au sein du service d'oto-rhino-laryngologie et chirurgie cervico-faciale de l'hôpital militaire Avicenne de Marrakech.

## Introduction

L'otite moyenne chronique cholestéatomateuse est une affection fréquente et grave. Elle met en jeu le pronostic fonctionnel auditif et le pronostic vital par l'exposition à des complications dangereuses justifiant le recours exclusif à la chirurgie. L’évolution récente des techniques chirurgicales et plus particulièrement l'apport de l'oto-vidéo-endoscopie et des nouvelles techniques d'imagerie médicale ont permis d'améliorer la prise en charge thérapeutique de cette pathologie ainsi que le suivi postopératoire. Le but de notre travail est d'analyser à travers une large revue bibliographique les particularités épidémiologiques, cliniques, paracliniques, thérapeutiques et évolutives des cholestéatomes de l'oreille moyenne.

## Méthodes

Notre travail est une étude rétrospective, réalisée au service d'oto-rhino-laryngologie et chirurgie cervico-faciale de l'hôpital militaire Avicenne de Marrakech, étalée sur une période de 11 ans, du premier janvier 2000 au 31 décembre 2010, et portant sur 145 cas de cholestéatome de l'oreille moyenne chez une population adulte. Les critères d'inclusion étaient les patients opérés pour otite moyenne chronique cholestéatomateuse. Nous avons exclu de cette étude les dossiers inexploitables et les otites chroniques non cholestéatomateuses, ainsi que les otites chroniques cholestéatomateuses chez l'enfant. Le recueil des données a été réalisé à l'aide d'une fiche d'exploitation où sont rapportées les données personnelles, cliniques, paracliniques, thérapeutiques et évolutives de chaque patient.

## Résultats

**Figure 1:**
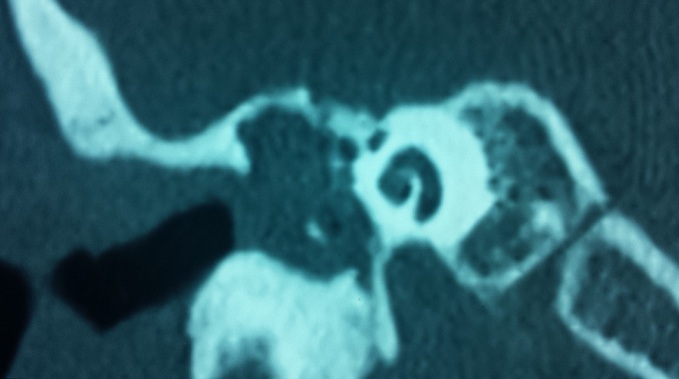
TDM des rochers en coupe coronale montant un cholestéatome de l'oreille moyenne avec lyse des osselets et érosion du mur de la logette

**Figure 2:**
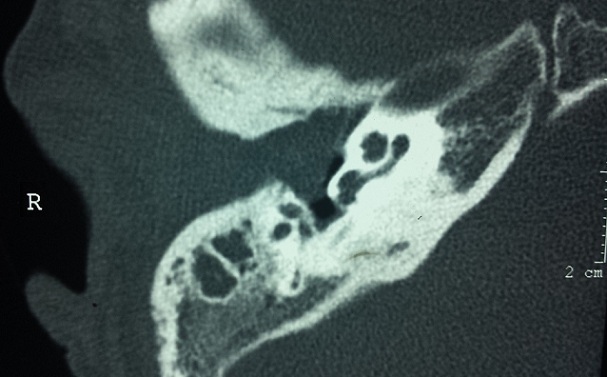
TDM des rochers en coupe axiale montrant un cholestéatome de l'oreille moyenne avec lyse complète de la chaine ossiculaire

La population étudiée a comporté 145 patients, dont 98 (67,6%) étaient de sexe masculin, soit un sexe ratio de 2. L’âge moyen de notre série était de 35 ans avec des âges extrêmes de 19 et de 70 ans. La tranche d’âge comprise entre 29 et 48 ans a été la plus touchée avec un taux de 42% des cas. Des antécédents d'intervention chirurgicale pour cholestéatome ont été retrouvés dans 32 cas (22%), d'otite à répétition dans 130 cas (89,6%), de rhino-sinusite chronique dans 35 cas (24,1%), et d'une fracture du rocher chez 2 cas (1,4%). Nous n'avons noté aucun antécédent familial de cholestéatome. La révélation clinique était une otorrhée fétide associée à une hypoacousie dans tous les cas (100%). Les complications étaient la révélation dans 29 cas (20%), dont 20 cas de mastoïdite (13.8%), 6 cas (4,1%) de paralysie faciale périphérique et 3 cas de labyrinthite (2%). La perforation marginale postéro-supérieure était la lésion la plus rencontrée à l'examen otoscopique (39%), suivie par la perforation atticale (20,7%) et de la poche de rétraction chez 22 patients (15,2%). L'audiogramme a été réalisé chez tous nos patients, ainsi que le scanner des rochers qui a montré une lyse partielle ou complète de la chaîne ossiculaire chez 117 cas (81%) et une érosion du mur de la logette chez 89 cas (62%) (Figure 1, Figure 2). La tympanoplastie en technique fermée a été faite chez 90 malades (62%) avec reconstruction par l'aponévrose dans 64 cas (44,1%), par le cartilage dans 16 cas (11%) et par les deux dans 10 cas (6.9%). La tympanoplastie en technique ouverte a été réalisée chez 46 patients (31.7%). L’épitympanotomie trans-canalaire a été faite dans 9 cas (6.6%) avec reconstruction systématique par du cartilage. L'ossiculoplastie de type II a été réalisée dans 38 cas (26,2%), alors que l'ossiculoplastie de type III a été réalisée dans 11 cas (7,6%). Parmi les 104 patients suivis, on a noté la survenue de 40 cas de récidive (38,4%) dont 26 cas chez des patients opérés par la technique fermée (25%) et 14 cas chez des patients opérés par la technique ouverte (13,4%).

## Discussion

Le cholestéatome se définit par la présence dans les cavités de l'oreille moyenne d'un épithélium malpighien kératinisant doté d'un potentiel de desquamation, de migration et d’érosion. On différencie l'otite chronique cholestéatomateuse acquise du cholestéatome congénital ou primitif. Ce dernier, beaucoup plus rare que l'otite cholestéatomateuse, correspond à l'absence de résorption des cellules embryonnaires épidermiques et se développe au début derrière une membrane tympanique intacte et le plus souvent en l'absence de pathologie inflammatoire muqueuse. Pour l'otite cholestéatomateuse acquise, sa pathogénie a été le sujet de plusieurs théories et dont les plus retenues sont la métaplasie de la muqueuse de l'oreille moyenne par un processus inflammatoire, la théorie de l'inclusion épidermique, la théorie de la migration latérale, la prolifération papillaire, et enfin la théorie de la poche de rétraction. Pour certains auteurs, l'otite cholestéatomateuse représente exclusivement l’évolution ultime d'une poche de rétraction. [1, 2]. Le cholestéatome acquis représente à peu près un tiers des otites moyennes chroniques suppurées. L’âge moyen pour certains auteurs est compris entre 35 et 43 ans, cela rejoint les résultats de notre série. La répartition selon le sexe est très variable dans la littérature [3–6].

L'otorrhée, surtout fétide, et l'hypoacousie sont les motifs de consultation les plus courants, ce qui rejoint nos résultats. Parfois, c'est une otorragie qui amène le patient à consulter. Dans certains cas, le cholestéatome est diagnostiqué à l'occasion de vertige, paralysie faciale, signes neurologiques ou fistulisation cutanée, témoignant d'une complication. Plus rarement, la découverte est fortuite lors d'un examen otoscopique, d'une imagerie ou d'une chirurgie sur l'oreille moyenne [5, 7].

L'examen otoscopique est la clé du diagnostic. Il doit être réalisé sous microscope après une aspiration minutieuse et suivi en cas de besoin d'un examen oto-vidéo-endoscopique. Le diagnostic est confirmé par la présence dans l'oreille moyenne de squames épidermiques émergeants d'une perforation marginale ou d'une poche de rétraction tympanique [2].

L'examen clinique doit comporter un testing musculaire facial, un examen mastoïdien, un examen neurologique et doit rechercher le signe de la fistule caractéristique d'une fistule labyrinthique. L′examen de l′oreille opposée est systématique. L′examen régional, rhinopharyngé, voire général permettra d′apprécier la présence ou non de facteurs étiologiques ou favorisant un état inflammatoire chronique. L'acoumétrie et l'audiométrie vont permettre d'objectiver une surdité de transmission, une surdité mixte du fait d'une atteinte labyrinthique associée ou parfois même une cophose [2]. Dans certains cas, le cholestéatome après avoir détruit les osselets peut se substituer à eux et rétablir l'effet columellaire, ce qui peut même positiver le Rinne, rendant nécessaire l'interprétation prudente de l'audiogramme.

L'imagerie moderne occupe actuellement une place prépondérante dans la prise en charge pré et postopératoire du cholestéatome de l'oreille moyenne. En pré-opératoire, un scanner des rochers est un examen quasiment incontournable pour le bilan d'extension, la recherche de certaines complications et pour la stratégie opératoire en objectivant les particularités anatomiques [7]. Parfois, il permet de confirmer le diagnostic dans les rares cas où l'examen otoscopique n'est pas suffisamment contributif. Les signes les plus évocateurs du cholestéatome sont la présence d'une masse tissulaire dans les cavités tympano-mastoïdiennes et d'une ou multiples zones d'ostéolyse, surtout au niveau de la chaîne ossiculaire et du mur de la logette. L'extension de l'ostéolyse vers le tegmen, le canal du facial, le labyrinthe, surtout le canal semi-circulaire latéral, témoigne de l'extension ou des complications du cholestéatome [2, 7]. L'imagerie par résonance magnétique peut parfois être indispensable pour le diagnostic et le bilan d'extension des complications encéphaliques [2]. En post-opératoire, le scanner reste l′examen de première intention, éventuellement complété par l'imagerie par résonance magnétique, surtout lorsqu'il existe un doute concernant la nature de l'opacité tissulaire à l'examen tomodensitométrique. Dans ce cas, l'IRM, avec des clichés tardifs en séquences T1 après injection de gadolinium ou avec séquence de diffusion, peut montrer une différence entre tissu cicatriciel fibro-inflammatoire et récidive de cholestéatome [2, 8].

Le traitement de l'otite chronique cholestéatomateuse est exclusivement chirurgical. Il doit éradiquer le cholestéatome et sa matrice, améliorer l'audition et enfin éviter au maximum la récidive qui reste le principal problème malgré l’évolution des techniques chirurgicales. Il existe classiquement deux grands types d'interventions en fonction de la conservation ou non du conduit osseux: la technique conservatrice du conduit osseux ou tympanoplastie en technique fermée, consistant en une masto-antro-atticotomie, avec le plus souvent une tympanotomie postérieure et/ou supérieure, et la technique avec sacrifice du conduit osseux ou tympanoplastie en technique ouverte avec ou sans comblement postérieur, dénommée encore cavité d’évidement pétro-mastoïdien [9]. A côté de ces deux techniques de base, il faut citer une intervention rarement utilisée, l’épitympanotomie trans-canalaire qui s'adresse à des petits cholestéatomes sacs, strictement limités à l'attique, tout le reste de l'oreille moyenne étant normal [2]. En effet, le choix de la technique dépend de nombreux paramètres: l’état de l′oreille malade et de l′oreille controlatérale, l'audition, les antécédents otologiques et généraux, le terrain naso-sinusien et la tomodensitométrie préopératoire. Toutefois, les indications opératoires sont l'objet d'une controverse intarissable opposant technique fermée - technique ouverte. Les tenants de la technique fermée restaurent l'anatomie normale de l'oreille moyenne et du conduit auditif externe ce qui permet de préserver une audition adéquate et libère des problèmes de soins post-opératoires, malgré le fait qu'elle expose à un plus grand risque de récidive. Les tenants de la technique ouverte créent un nouveau statut anatomo-physiologique de l'oreille afin d'en modifier les conditions locales qui ont engendré la maladie cholestéatomateuse (Tableau 1).

**Tableau 1:** Traitement chirurgical du cholestéatome suivant le choix des techniques

Série	TTF	TTO
Gaillardin (16)	100%	0%
Hasbellaoui (17)	94%	6%
Chakroun (25)	3%	97%
Abada (24)	20%	80%
Notre série	62%	31,7%

TTF: tympanoplastie en technique fermée; TTO: tympanoplastie en technique ouverte

Actuellement, quoique la plupart des auteurs privilégient la technique conservatrice, la tympanoplastie en technique ouverte a encore sa place, en particulier lorsqu'un deuxième temps chirurgical n'est pas possible ou lorsqu'il y a impossibilité d'un suivi rigoureux ou encore en cas de récidives. L'indication d'un deuxième temps opératoire n'est plus systématique pour 2 raisons: l'utilisation de plus en plus importante de l'oto-vidéo-endoscopie qui a diminué le risque de cholestéatome résiduel en contrôlant la totalité de l'exérèse dans des zones d'accès difficiles, et l’évolution de l'imagerie permettant la sélection des indications de ces reprises chirurgicales [2]. La réhabilitation de l'audition dans la chirurgie du cholestéatome fait appel soit aux différents types d'ossiculoplastie, réalisés dès le premier temps opératoire si la muqueuse paraît saine, soit aux prothèses auditives conventionnelles ou à ancrage osseux.

Malgré le développement des techniques opératoires et des moyens de l'oto-endoscopie et de l'imagerie, les récidives représentent encore un véritable problème de la chirurgie des cholestéatomes, rendant nécessaire une surveillance rigoureuse et étroite de tout patient opéré. Malheureusement, on rencontre encore dans la littérature un taux non négligeable de perdus de vue allant jusqu′à 50% après 2 ans de suivi [10]. Dans notre étude, 28% de patients ont été perdus de vue sur une période de 11ans, 26 cas de récidive ont été notés après une technique fermée et 14 cas de récidive après une technique ouverte [1] (Tableau 2).

**Tableau 2:** Fréquences de récidive cholestéatomateuse selon la technique

Récidive		
Série	TTF	TTO
Sheehy (12)	51%	51%
Smyth (13)	8,5%	1%
Brown (14)	34%	11%
Notre série	25%	13,46%

TTF: tympanoplastie en technique fermée; TTO: tympanoplastie en technique ouverte

## Conclusion

L’étude de notre série de 145 cas associée à une revue de la littérature confirme la fréquence et la gravité de l'oreille moyenne cholestéatomateuse, l'intérêt de la clinique dans le diagnostic et surtout la place incontournable de l'imagerie moderne dans le bilan pré-opératoire et dans la surveillance post-opératoire, permettant souvent de se passer d'un temps de révision chirurgicale. Enfin, le choix plus réglé et mieux codifié de la technique chirurgicale, ainsi que l'introduction systématique des optiques d'oto-endoscopie permettent de minimiser le taux de cholestéatome résiduel et de récidive.
